# Juvenile methylphenidate reduces prefrontal cortex plasticity via D3 receptor and BDNF in adulthood

**DOI:** 10.3389/fnsyn.2014.00001

**Published:** 2014-01-21

**Authors:** Susan L. Andersen, Kai C. Sonntag

**Affiliations:** ^1^Laboratory for Developmental Neuropharmacology, McLean Hospital and Harvard Medical SchoolBelmont, MA, USA; ^2^Department of Psychiatry, McLean Hospital and Harvard Medical SchoolBoston, MA, USA

**Keywords:** ADHD, adolescence, child, methylphenidate, ritalin, sensitive period

## Abstract

**Background**: Early drug intervention in childhood disorders aims to maximize individual potential in the short- and long-term. Consistently, juvenile exposure to psychostimulants, such as methylphenidate (MPH), reduces risk for substance use in animals and sub-populations of individuals with attention deficit hyperactivity disorder (ADHD). We investigated the effects of MPH on brain plasticity via dopamine receptor D3 (D3R) and brain-derived neurotrophic factor (BDNF) expression in developing rats.

**Methods**: Between postnatal days 20–35, rat pups were administered saline vehicle (Veh) or MPH (2 mg/kg), the D3R-preferring agonist ±7-OHDPAT, or the antagonist nafadotride (0.05 mg/kg) alone, or in combination with MPH twice a day. In adulthood, subjects were challenged to Veh or cocaine (10 mg/kg for two days). The prefrontal cortex was analyzed for protein and mRNA levels of total BDNF, its splice variants I, IIc, III/IV, and IV/VI, and D3 receptors. A separate group of subjects was assessed for splice variants at 20, 35, 40, and 60 days of age.

**Results**: Across age strong correlations were evident between *Drd3* and *Bdnf* mRNA levels (*r* = 0.65) and a negative relationship between *Drd3* and exon IIc after MPH treatment (*r* = −0.73). BDNF protein levels did not differ between Veh- and MPH subjects at baseline, but were significantly lower in MPH-treated and cocaine challenged subjects (30.3 ± 9.7%). *Bdnf* mRNA was significantly higher in MPH-treated subjects, and reversed upon exposure to cocaine. This effect was blocked by nafadotride. Furthermore, *Bdnf*_total_ and *Bdnf* splice variants I, IIc, III/IV, and IV/VI changed across the transitions between juvenility and late adolescence.

**Conclusions**: These data suggest a sensitive window of vulnerability to modulation of BDNF expression around adolescence, and that compared to normal animals, juvenile exposure to MPH permanently reduces prefrontal BDNF transcription and translation upon cocaine exposure in adulthood by a D3R-mediated mechanism.

## Introduction

Children with Attention Deficit Hyperactivity Disorder (ADHD) often respond favorably to psychostimulant therapy, contributing to its use by 61% of children with ADHD between the ages of 6–13 years (Dalsgaard et al., 2013). The most commonly prescribed stimulant is methylphenidate (MPH). MPH predominantly affects the dopamine and noradrenergic systems, with recent evidence showing a role of the dopamine D3 receptor (D3R) in ADHD-associated behaviors in animals (Andersen et al., 2008; Barth et al., 2013). For example, D3R antagonists reduce hyperactivity and facilitate object recognition in DAT knockout mice, which have been used to model an ADHD phenotype. Other studies further localize D3R actions in object recognition to the prefrontal cortex (Watson et al., 2012).

Childhood/juvenile exposure to psychostimulants during this time of elevated plasticity produces effects that are opposite of those observed in drug-exposed adult animals (Brandon et al., 2001; Dow-Edwards and Busidan, 2001; Andersen et al., 2002; Bolanos et al., 2003; Andersen, 2005). For example, juvenile, but not adult, exposure to cocaine reduces drug seeking later in life (Dow-Edwards and Busidan, 2001; Andersen et al., 2002). Similarly, juvenile MPH decreases *Drd3* mRNA in the prefrontal cortex (PFC) (Andersen et al., 2008), in contrast to an increase in the same region in adult, stimulant-exposed animals (Le Foll et al., 2005). While most adult studies have focused on D3R changes in the nucleus accumbens (Everitt and Robbins, 2000; Le Foll et al., 2005), the reduction in D3R following juvenile MPH exposure is not evident in that region (Andersen et al., 2008). Rather, the juvenile MPH effect on D3R and an MPH-induced place aversion was recapitulated by juvenile treatment with the D3R agonist ±7-OHDPAT (Andersen et al., 2008). Finally, microinjections of ±7-OHDPAT into the PFC reversed aversion, resulting in a preference for cocaine-associated environments.

Cue responsiveness to drug-associated contexts depends on neuroplasticity associated with brain-derived neurotrophic factor (BDNF) levels in adult animals within the prelimbic (pl) PFC (Berglind et al., 2007). Adult BDNF levels are transiently elevated following an acute injection of cocaine in both the PFC and the nucleus accumbens (NAc), with sustained elevations of *Bdnf* and *Drd3* mRNA found 60 days later in the NAc (Le Foll et al., 2005). Juvenile exposure to MPH reduces *Bdnf* mRNA in the striatum and the hypothalamus with no change in the cingulate cortex during peri-adolescence (Chase et al., 2007). Similar findings of no change in *Bdnf* mRNA were evident in the ventral tegmental area both immediately after treatment and long-term (Warren et al., 2011). However, the enduring effects of MPH exposure interact with development to manifest fully later in life (Andersen, 2005; Brenhouse and Andersen, 2011). Therefore, the lack of changes in BDNF expression as observed by Chase et al. (2007) in the PFC and Warren et al. (2011) in the ventral tegmental area could have been undetectable, because the window of observation was too early. In order to investigate the time course of D3 receptors and BDNF expression in the developing PFC, we examined the postnatal expression of these two indices and their inter-relationship (Experiment 1). We also examined how previous exposure to MPH may or may not modify BDNF levels to cocaine later in life (Experiment 2). As we suspect that D3 receptors modify BDNF expression in the medial PFC, we manipulated D3 activity *in vivo* using juvenile exposure to MPH, ±7-OHDPAT, and the D3R antagonist nafadotride (Experiment 3). Ultimately, we were interested in how juvenile exposure to MPH or a D3R-preferring agonist modulates BDNF expression later in life at baseline and following a 2-day unbiased place conditioning paradigm to 10 mg/kg cocaine.

Environmental influences, such as drug exposure during sensitive periods, can further modulate synaptic structure by altering the expression of *Bdnf* splice variants (Boulle et al., 2012). Such specific modulation of splice variants permits both spatial and temporal regulation of BDNF expression that in turn, can lead to the precise building of synaptic structure and respond to environmental demands. Understanding how specific splice variants of the *Bdnf* gene change across age may provide insight into the nature of maturation of different parts of the neuron. Exon I and II are specific to neurons, whereas exons III and IV are found in non-neural tissue as well (Nakayama et al., 1994). The exon I splice variant is expressed primarily in the soma and dendrites, whereas exon II is predominantly in the dendrites, and exon IV is restricted to the soma (Boulle et al., 2012).

Empirical data suggest that different exons are altered following experiences in a semi-unique manner. For example, exon II seems to be preferentially regulated in reward circuits (McCarthy et al., 2012). Sadri-Vakili et al. (2010) demonstrated that chronic cocaine increased *Bdnf* exon IV in the PFC of adult rats. Exon IV contains the binding sites for CREB and MeCP2 and plays a role in cognitive processes and learning and memory—such as those required for encoding drug-associated cues (Boulle et al., 2012; McCarthy et al., 2012). While BDNF changes have already been associated with addiction-related alterations in structure and function, the majority of these studies have been performed following adult drug exposure paradigms. We are particularly interested in whether exon IV changes following juvenile exposure to MPH, which we have shown increases CREB expression (Andersen et al., 2002). To investigate whether stimulants have unique effects on *Bdnf* splice variants, we determined how *Bdnf*_total_, and different 5' exons splice variants (exons I, IIc, III/IV, and IV/VI), change across age within the late maturing PFC (Experiment 4). Here, we used quantitative Real-Time PCR (qRT-PCR) to examine changes in *Bdnf* exons, as protein-labeling antibodies for the specific exons are not yet available.

## Materials and methods

### Subjects

**Figure 1:**
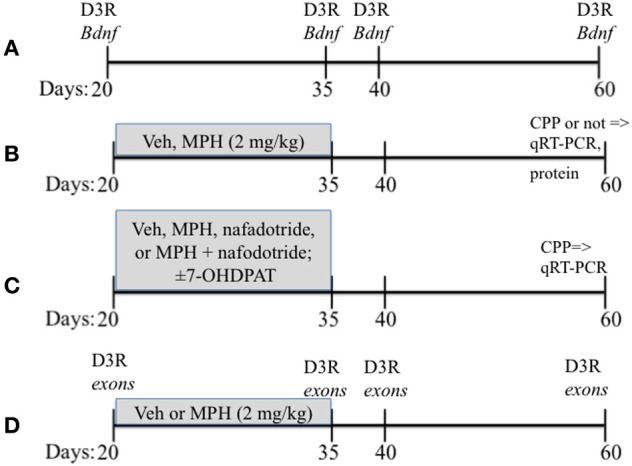
**A schematic showing the overall experimental design that includes the timing of drug treatments**. Vehicle (Veh), methylphenidate (MPH), conditioned place preferences (CPP). Ages of rats are in days. **(A–D)** presents a schematic of the paradigms used for Experiments 1–4.

Lactating female Sprague-Dawley rats obtained from Charles River were housed with their litters on a 12 h/12 h light/dark cycle with lights on at 07:00 h with food and water provided *ad libidum*. Litters were culled to 8 pups of equal numbers of males and females on postnatal day 1 (P1), and were weaned at P21. Care was taken to distribute each condition to one male from each litter. Only males were used for these studies, with the remaining littermates used in other studies. The overall experimental design is found in Figure 1.

### Drugs

MPH HCl (d,l-MPH), ±7-OHDPAT, nafadotride, and cocaine HCl were obtained from Sigma (St. Louis, MO). Drugs were dissolved in 0.9% saline (vehicle) and administered in a volume of 1 ml/kg. Our earlier study demonstrated no behavioral difference with i.p. vs. oral administration in place conditioning (Brenhouse et al., 2009).

### qRT-PCR

qRT-PCR was performed as described (Andersen et al., 2008). Briefly, brains were rapidly dissected into the mPFC. Sections were snap-frozen in TriReagent solution (Sigma) and stored at −80°C until further analysis. Samples were homogenized, total RNA extracted, DNAse digested (Ambion, Austin, TX), and reverse transcribed into cDNA using the SuperScript II First-Strand Synthesis System (Life Technologies, Foster City, CA). cDNA samples were analyzed by qPCR using the iQ SYBR Green Supermix (BioRad). Amplifications were performed in a total volume of 20 μl with 40 nMol primers for each reaction on an Opticon MJ Thermocycler; MJ Research (Watertown, MA). The following published primer sequences were used: *Bdnf* exons I, II, II, and (Tsankova et al., 2006) *Bdnf*_total_ (Asai et al., 2007), and *Drd3* and *Gapdh* (Andersen et al., 2008).

*Bdnf*_total_: F: 5′-ACTCTGGAGAGCGTGAATGG-3′ R: 5′-TACTGTCACACACGCTCAGC-3′
Exon I: F: 5′-CCTGCATCTGTTGGGGAGAC-3′ R: 5′-GCCTTGTCCGTGGACGTTTA-3′
Exon II: F: 5′-CTAGCCACCGGGGTGGTGTAA-3′ R: 5′-AGGATGGTCATCACTCTTCTC-3′
Exon III F: 5′-CTTCCTTGAGCCCAGTTCC-3′ R: 5′-CCGTGGACGTTTACTTCTTTC-3′
Exon IV F: 5′-CAGAGCAGCTGCCTTGATGTT-3′ R: 5′-GCCTTGTCCGTGGACGTTTA-3′
*Drd3*: F: 5′-AAGCGCTACTACAGCATCTGC-3′ R: 5′-GGATAACCTGCCGTTGCTGAG-3′
*Gapdh*: F: 5′-AACTCCCATTCTTCCACCTTTG-3′ R: 5′-CCCTGTTGCTGTAGCCATATTC-3′

The *Bdnf* exon primers were verified by sequence alignment using deposited sequences in GenBank: Exon I, transcript variant I (Accession EF125675); Exon II: transcript variant IIc (Accession EF125678); Exon III: transcript variant III (Accession EF125686); Exon IV: transcript variant IV (Accession EF125679). A new nomenclature for *Bdnf* transcript variants has recently been suggested by Aid et al. (2007). Accordingly, our primer sequences for exon II, III, and IV amplify new exons IIc, IV, and VI, respectively. Thus, for each exon both the old and the new nomenclature are indicated.

### Design

#### Experiment 1: the relationship between Drd3 and *Bdnf* mRNA *in vivo*

***Subjects and Design***. mRNA from the PFC was assessed to determine the nature of the relationship between *Bdnf*_total_ and *Drd3* across age. Rats were sacrificed at 4 ages: P25, 35, 40, and 60 representing juvenile, early adolescent, mid adolescent and late adolescent stages respectively (Figure 1A). The PFC was rapidly (<1 min) dissected out onto chilled glass plates. The sample was then processed for qRT-PCR as described in detail above (3.3). An *n* = 6 for P25 and *n* = 5 for P35, 40, and 60 were used.

#### Experiment 2: measurement of *Bdnf*_total_ mRNA and Bdnf protein levels following MPH treatment with and without cocaine conditioning

***Drug treatment paradigm***. Subjects were weighed daily at 09:00 h and injected with either MPH (2 mg/kg) or saline vehicle (Veh) between P20-35 (see Figure 1B; Andersen et al., 2002, 2008. The second injection was administered 4 h later at 13:00 h. The dose of MPH was selected on the basis of previous studies (Andersen et al., 2002), and approximates a clinically relevant dose in humans based on plasma levels (Wargin et al., 1983; Kuczenski and Segal, 2002). After P35, the rats received no further drug treatment until behavioral testing or sacrifice for the pharmacological studies 25 days later at P60.

***Design***. To determine whether PFC BDNF levels and MPH exposure interacted with place conditioning to cocaine, we examined mRNA and protein levels. Drug-exposed subjects were housed with littermates and grown to P60, when they either (1) remained naïve to cocaine exposure (cocaine-naïve) or 2 underwent place conditioning to cocaine (10 mg/kg, i.p.). The 10 mg/kg dose of cocaine was chosen based on prior studies (Andersen et al., 2002; Carlezon et al., 2003) that demonstrated a clear differentiation of place conditioning effects between Veh animals. Our earlier study with these animals showed that the Veh subjects demonstrated a minimal preference for cocaine-associated environment, whereas the MPH animals demonstrated a significant place aversion (Andersen et al., 2008). Two sets of subjects were run: one set for *Bdnf* mRNA (*n* = 6) and the second set for BDNF protein (*n* = 5).

***Cocaine place conditionin***. Unbiased place conditioning, according to standard laboratory methods (Andersen et al., 2002; Carlezon et al., 2003), occurred in a 3-chamber apparatus. This chamber consisted of two large (24 × 18 × 33) side compartments separated by a small (12 × 18 × 33 cm) middle compartment. Screening was conducted for 30 min on day 1. Rats were placed in the middle compartment and allowed to freely explore the apparatus. Rats that demonstrated a clear preference for either side (>18 of 30 min) were eliminated from further testing. Two days of conditioning (with 2 sessions per day) occurred on day 2 and 3 for 60 min, and robust conditioning under these conditions was observed (Andersen et al., 2002; Carlezon et al., 2003). During the conditioning sessions, rats received a 1 ml/kg, i.p. injection of Veh in the morning (09:00 h) and confined to one side. The animals were then returned to the home cage. Four hours later, rats received 10 mg/kg cocaine and confined to the other side. The dose of cocaine for the place conditioning experiments was based on previous results that demonstrated consistent place aversion in MPH-exposed male rats at P60 (Andersen et al., 2002; Carlezon et al., 2003). Sides differed in floor texture, wall colors, and lighting, and assignments were counterbalanced. On day 4, rats were permitted to freely explore the entire apparatus for 30 min in a drug-free state.

***BDNF protein immunoassays***. BDNF protein was extracted from the PFC. Dissected tissue was weighed and homogenized in 10 vol/wt of cold extraction buffer consisting of: 100 mM Tris/HCl, ph = 7.0, 2% bovine serum albumin, 4 mM EDTA, 2% Triton-X, and the protease inhibitors aprotinin (5 μg/ml), benzamidine HCl (157 μg/ml), pepstatin A (0.1 μg/ml), and phenylmethylsulfonyl fluoride (PMSF; 17 μg/ml). Homogenates were centrifuged at 14,000 rpm for 30 min at 4°C and the supernatant was used in the assay. BDNF protein was quantified using the ChemiKine BDNF Sandwich ELISA kit (Chemicon, Temecula, CA) according to the manufacturer's directions. Samples were run in duplicate and quantified using standards run simultaneously with the experimental samples.

#### Experiment 3: relationship between *Bdnf*_total_ mRNA and D3R drug treatment

***Subjects***. Subjects were exposed to ±7-OHDPAT (0.3 mg/kg; *n* = 6) between P20-35 or Veh (*n* = 6; Figure 1). To further confirm that the effects of MPH were mediated by the D3 receptor, a separate group of subjects was treated with MPH (2 mg/kg, i.p.; *n* = 6), nafadotride (0.05 mg/kg; *n* = 6), a combination of MPH and nafadotride (MPH/Nafad; *n* = 6) twice daily between the ages of P20-35 (Andersen et al., 2002).

***Design***. Subjects were tested with place conditioning to environments associated with 10 mg/kg cocaine at P60 (Andersen et al., 2008) and sacrificed 24 h after the last cocaine injection, the PFC rapidly dissected out, and *Bdnf* mRNA changes determined by qRT-PCR (Figure 1C).

#### Experiment 4: age and drug effects on the *Bdnf* splice variants exons I, IIc, III/IV, IV/VI and Drd3

***Subjects***. Rats the ages of 20, 35, 40, and 60 days were used in these studies (*n* = 6/age). The 20 and 35 days ages were selected to characterize the expression of exons during the drug exposure period, 40 and 60 days represent short- and long-term ages for the paradigm.

***Design***. The PFC was dissected and processed for mRNA for exons I, IIc, III/IV, IV/VI, and Drd3 across age. *Bdnf* exons were assessed from subjects exposed to MPH or Veh as juveniles (Figure 1D).

### Data analysis

The expression of the *Bdnf* exons was normalized to the housekeeping gene product *Gapdh*. Linearity (*r* = 0.9 or better) and detection limit of the assay were determined in successive 10-fold serial dilutions, performed in triplicate, and an optimal amount of template was chosen for the quantitative analysis. Quantification was performed at a threshold detection line (“threshold cycles,” *Ct* value). The Ct of each gene was normalized against *Gapdh*, which was run simultaneously for each marker. Expression levels and differences between treatment and Veh groups were determined according to the 2^−Δ Ct^ or 2^−ΔΔ Ct^ method, respectively (Livak and Schmittgen, 2001). Comparisons were made between drug groups with a two-tailed Student's *t*-test or an ANOVA (SPSS); statistical significance was set at *p* < 0.05. Data were expressed as mean ± s.e.m.

## Results

### Experiment 1: the relationship between Drd3 and *Bdnf* mRNA *in vivo*

**Figure 2:**
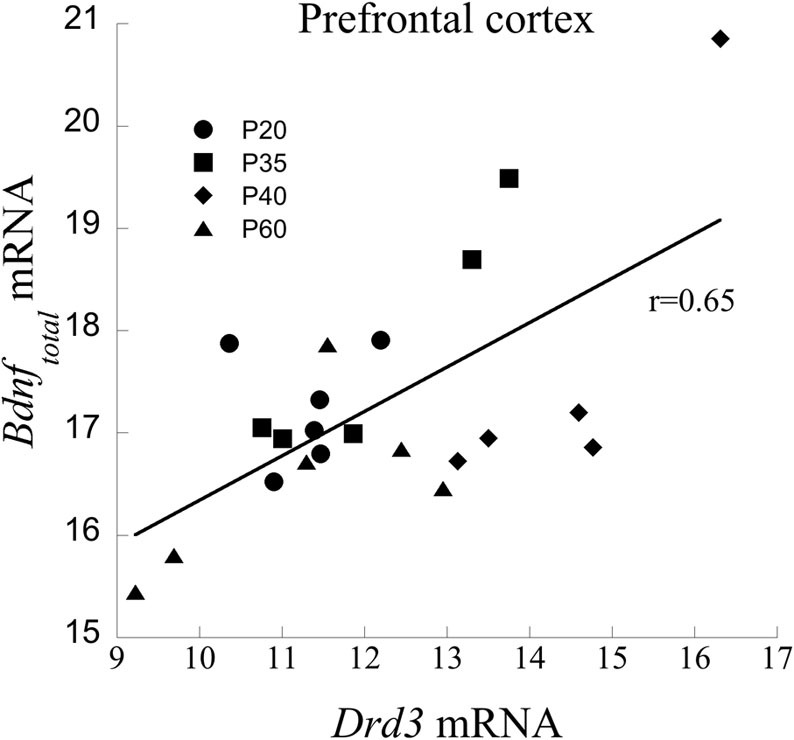
**Correlation between Drd3 and *Bdnf* mRNA from the PFC of developing rats at the ages of 20, 35, 40, and 60 days**.

mRNA from the PFC across age of animals was measured to determine a relationship between *Bdnf*_total_ and *Drd3*. Age itself had a significant effect on *Drd3* [*F*_(3, 17)_ = 7.87, *P* < 0.001], but did not significantly influence *Bdnf* mRNA (*p* = 0.1). Regression analysis with ANCOVA showed that *Drd3*, when co-varied for age, significantly predicted *Bdnf* mRNA [*F*_(1,19)_ = 18.20, *P* < 0.001] and accounts for 49% of *Bdnf* changes (Figure 2).

### Experiment 2: *Bdnf*_total_ mRNA and protein levels following MPH treatment

**Figure 3:**
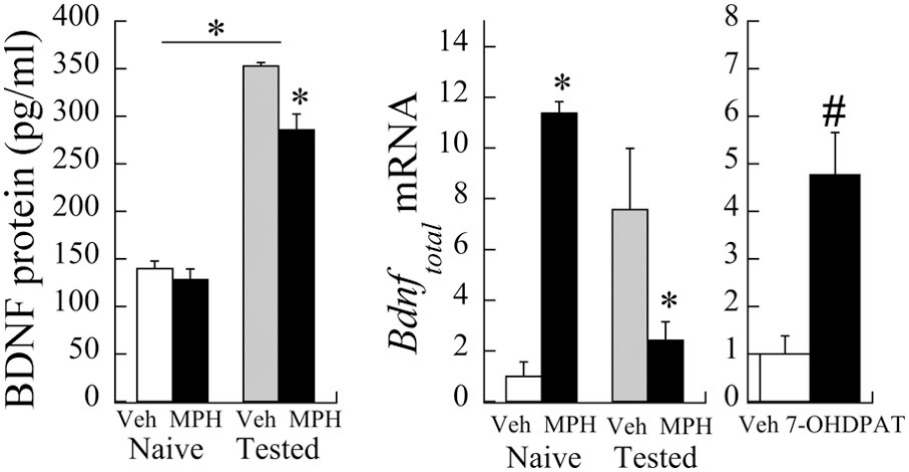
***Bdnf* mRNA changes following juvenile MPH treatment between 20 and 35 days of age relative to controls. (left)** Veh or MPH rats were assessed at P60 without prior cocaine place conditioning (naïve) or after place conditioning to 10 mg/kg cocaine for 2 days (tested). ^*^p < 0.05. **(right)** A separate group of subjects was exposed to the D3R-preferring agonist ±7-OHDPAT between P20-35 and assessed for *Bdnf* at P60. #*p* < 0.06 Mean ± *SE* for *n* = 6 subjects shown.

The relationship between PFC *Bdnf* mRNA levels and MPH exposure interacted significantly with whether or not the animals were behaviorally tested with cocaine [Exposure × Testing interaction: *F*_(1,12)_ = 6.01, *P* < 0.05]. *Bdnf* mRNA levels were elevated in MPH subjects relative to Veh controls, and this relationship reversed following behavioral testing to cocaine at P60 [Figure 3 (left)]. Similar to the effects of MPH exposure, juvenile treatment with the D3R-preferring agonist ±7-OHDPAT also increased *Bdnf* mRNA [Figure 3 (right)] in behaviorally-naïve subjects.

As observed in the mRNA levels, a significant interaction was observed in BDNF protein levels as a result of cocaine testing [Exposure × Testing interaction: *F*_(1,29)_ = 5.35, *p* < 0.05]. Baseline levels of BDNF protein (pg/ml) did not significantly differ between MPH and Veh subjects [*t*_(8)_ = 0.83, *p* = 0.4; see Figure 3). After cocaine administration at P60, however, BDNF protein levels in Veh subjects increased 253.1 ± 28.9% relative to cocaine naïve controls. In contrast, MPH-exposed subjects had a slightly smaller percent increase (222.2 ± 24.4%) relative to cocaine naïve levels, consistent with lower *Bdnf* mRNA levels in this group.

### Experiment 3: relationship between *Bdnf*_total_ mRNA, D3R, and MPH treatment

**Figure 4:**
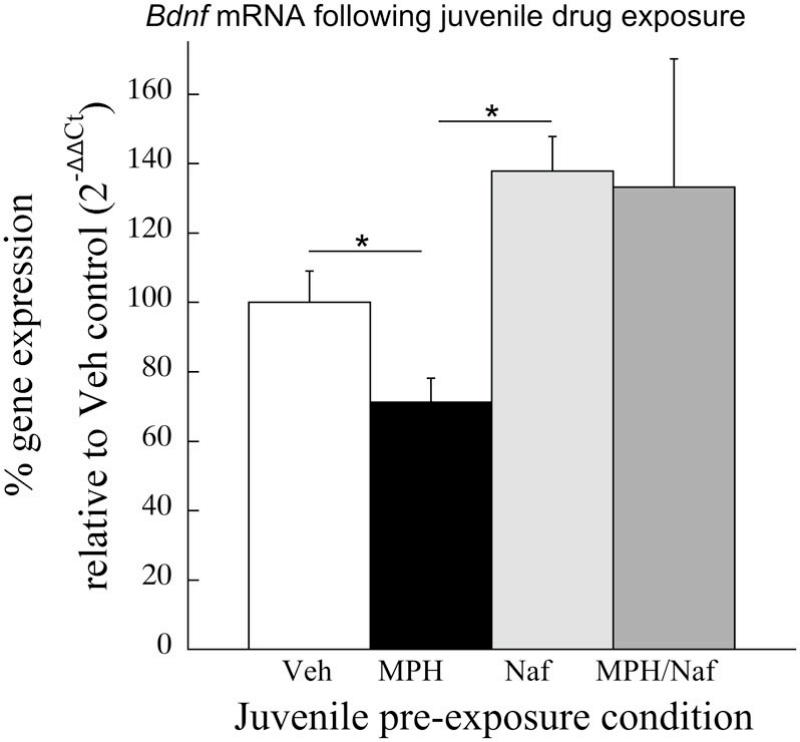
**The effects of juvenile treatment with Veh, MPH, the D3R antagonist nafadotride or MPH in combination with nafadotride on *Bdnf* mRNA levels in the PFC following place conditioning**. Means ± *SE* are presented for *n* = 7–8 subjects/condition. ^*^indicates significant difference at *p* < 0.05.

Consistent with the data in Experiment 2, following juvenile MPH exposure and place conditioning to cocaine, *Bdnf* mRNA levels were significantly reduced 30.3 ± 9.7% when compared to the Veh exposed subjects [*t*_(11)_ = 2.69, *p* < 0.03; Figure 4]. In contrast, *Bdnf* mRNA significantly increased 35% following juvenile exposure to the D3R antagonist, nafadotride, when compared with Veh controls [*t*_(12)_ = 2.65, *p* < 0.03]. No significant difference was observed between the Veh and the MPH/Nafad subjects [*t*_(13)_ = 0.74, *p* > 0.4].

### Experiment 4: age and drug effects on *Bdnf* splice variants I, IIc, III/IV, IV/VI

#### Bdnf mRNA exons change across age

**Figure 5:**
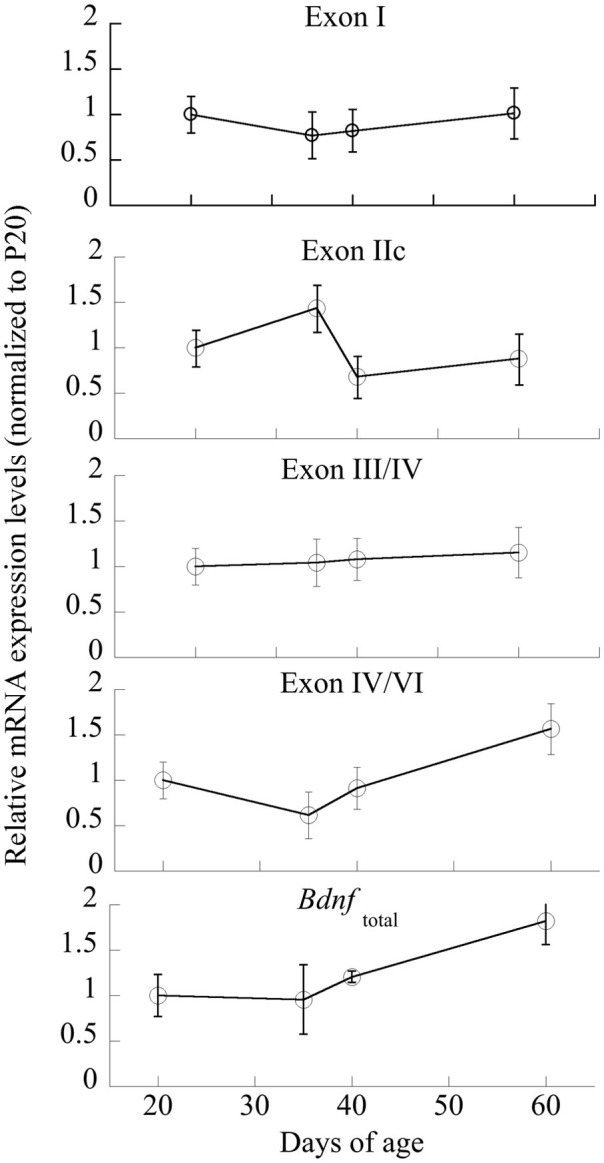
**Changes in mRNA expression of *Bdnf* and its exon-specific splice variants I, IIc, III/IV, and IV/VI as a function of age**. Data are expressed following correction for GAPDH levels run simultaneously. *n* = 5–6/age, with Means ± *SE* presented.

Individual ANOVAs of exons I, IIc, III/IV, IV/VI were run to determine significant changes across Age; only exon IIc and IV/VI were significantly different [*F*_(3,20)_ = 3.35 and 4.45, respectively, *p* < 0.05; Figure 5]. Exons I and III/IV did not significantly change across maturation (*p* > 0.6), nor did *Bdnf*_total_ (*p* < 0.14).

#### Bdnf exon IIc and IV/VI mRNA, Drd3, and MPH exposure

**Figure 6:**
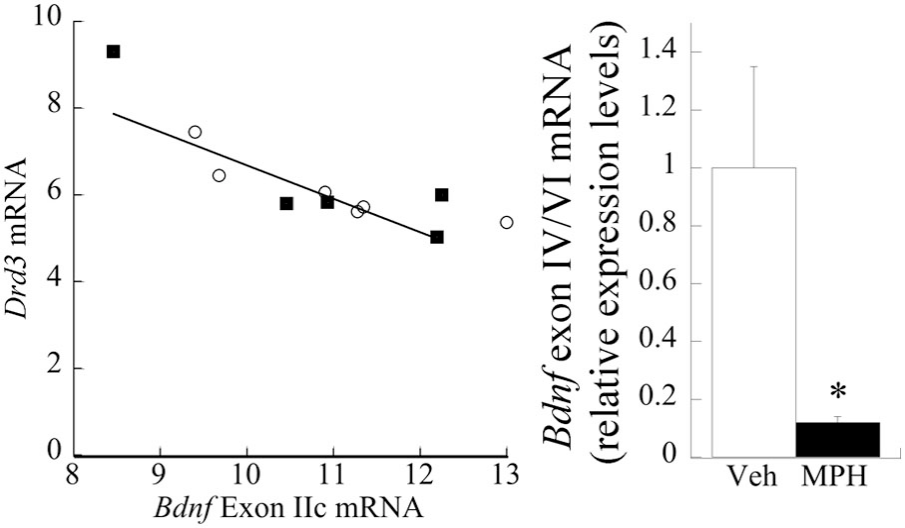
**Relationship between D3R, MPH, and BDNF exon splice variants. (left)** *Drd3* mRNA negatively correlates (Pearson's *r* = −0.73) with exon IIc expression. The filled squares represent the MPH subjects at P60, whereas the open circles are Veh. **(right)** Juvenile treatment with MPH significantly reduces exon IV/VI expression at 60 days of age relative to the Veh. *n* = 6/group, with Means ± *SE* presented. ^*^p < 0.05.

No significant main effect of MPH exposure was evident at P60 (*p* > 0.7) for exon IIc. However, a strong correlation existed between *Drd3* and exon IIc [Pearson's *r* = −0.73, *p* < 0.01; Figure 6 (left)]. Exon IV/VI was significantly reduced following MPH exposure [*F*_(1,10)_ = 42.43, *P* < 0.001], as shown in Figure 6 (right), however, this relationship did not correlate with changes in D3 receptors.

## Discussion

Our data show that juvenile exposure to MPH or manipulation of the D3 dopamine receptor has enduring effects on the expression of BDNF that are related to D3 receptor changes. D3R activation during development did not significantly change BDNF protein levels, but increased *Bdnf*_total_ mRNA while decreasing its splice variants IIc and IV/VI in adulthood. A negative correlation between *Drd3* and exon IIc mRNA expression was observed. In contrast, a reduction in *Bdnf*_total_ mRNA and protein were evident in CPP tested animals following D3 receptor activation during development.

*Bdnf* gene regulation occurs in response to environmental stimuli. As D3R antagonism attenuates cue-induced drug-seeking behavior in adult animals (Gal and Gyertyan, 2005), the observed decrease in *Drd3* mRNA is consistent with a reduction in place preferences for cocaine-associated cues in animals exposed to MPH during the juvenile period (Andersen et al., 2002). More importantly, changes in *Bdnf* and its association with *Drd3* suggest that the long-term plasticity observed in addiction (Sokoloff et al., 2001) is reduced in MPH animals. Clinically, a subtype of children that is treated with MPH shows reduced substance use disorder (Mannuzza et al., 2008), suggesting that BDNF may play a role.

A sensitive period exists for manipulations of PFC development, with pre-pubertal exposure to psychostimulants offering a window of opportunity for reduced substance abuse disorders (SUD; Andersen, 2005). D3 receptors are localized on dopamine terminals and non-terminal fields (Stanwood et al., 2000), which continue to develop into adolescence (Andersen et al., 2008). During adolescence, regulatory processes in the PFC change markedly (Andersen et al., 1997; Dumont et al., 2004; Tseng and O'Donnell, 2007) that lead to an increase in dopamine function. One of these effects is the loss of a dopamine auto-regulatory-type process mediated by D3 receptors (Booth et al., 1994; Andersen et al., 1997). The current data show that D3R changes across age account for 49% of the changes in *Bdnf* mRNA, suggesting that D3R may differentially sculpt the pre- vs. post-adolescent PFC by altering neurotrophic expression. Relative to controls, MPH-exposed animals show a significant increase in *Bdnf*_total_ mRNA, but no changes in protein at baseline without challenge. However, MPH animals had a reduction of *Bdnf*_total_ mRNA and protein following cocaine conditioning. BDNF is released with dopamine in the PFC in response to drug cues (Altar et al., 1998; McCarthy et al., 2012). The adult literature shows that cocaine-induced increases of BDNF suppress inhibitory GABAergic interneuron activity in the mPFC [resulting in increased long-term potentiation (LTP) (Lu et al., 2010)]. By extrapolation, a decline in BDNF following juvenile MPH and drug challenge in adulthood would reduce cortical output in response to drug cues relative to controls. A recent study shows that juvenile MPH reduces the number of spikes in PFC neurons in adulthood—consistent with this hypothesis (Urban et al., 2012).

Modulation of BDNF occurs by histone acetylation or post-transcriptional regulation (Mellios et al., 2008; Caputo et al., 2011; Boulle et al., 2012; Leal et al., 2014), and our data provide evidence for epigenetic modifications in BDNF splice variants by MPH or D3R. In development, the splice variants II and IV are typically decreased during the transition from juvenile to adolescence. These exons were also reduced in adult animals after juvenile MPH exposure. A negative correlation between exon IIc and *Drd3* mRNA suggests less neuronal plasticity as D3 receptors decrease either following typical aging or that facilitated by juvenile MPH treatment (Andersen et al., 2008). Exon II has been localized to neurons and its expression is activity-dependent in visual cortex (Pattabiraman et al., 2005), supporting a potential role in modulating neuronal plasticity. Exon IV/VI was also significantly lower in MPH subjects consistent with previous observations in juvenile MPH rats studied by Fumagalli et al. (2010). It should be noted that the observed up-regulation of *Bdnf*_total_ mRNA in adult animals after juvenile treatment with MPH did not correlate with BDNF protein levels. There is emerging evidence that protein expression is regulated on the post-transcriptional level by small non-coding RNAs, including micro (mi) RNAs. Several miRNAs have been identified that target the *Bdnf* transcript (Mellios et al., 2008; Caputo et al., 2011), indicating that other epigenetic mechanisms may be involved in regulating BDNF expression.

The enduring effects in juveniles are predictably opposite to the increase in *Bdnf* exon IV/VI expression in the PFC of adult, cocaine self-administering rats that may underlie craving and relapse (Sadri-Vakili et al., 2010; Schmidt et al., 2013). Exon IV/VI contains the binding sites for CREB and MeCP2 and plays a role in synaptic plasticity, cognitive processes and learning and memory—such as those required for encoding drug-associated cues (McCarthy et al., 2012). While earlier studies have demonstrated reduced cue responsiveness in rats exposed to MPH during the juvenile period (Andersen et al., 2002; Carlezon et al., 2003; Argento et al., 2012), others have shown that peri-adolescent treatment with MPH reduces object recognition later in life (Leblanc-Duchin and Taukulis, 2007). Interestingly, object recognition increases following D3 receptor blockade in the adult PFC (Watson et al., 2012). Together, these data suggest that MPH may also permanently modulate object recognition later in life. Object recognition measures whether a novel object is recognized, as new or not, which is impaired in ADHD.

Changes in BDNF are important during sensitive period when the programming of drug-seeking behavior occurs (Andersen et al., 2002; Andersen, 2005). Further understanding of the molecular mechanisms regulating these changes can potentially lead to novel ways of harnessing developmental relationships to redirect an off-course trajectory. For example, Shaw et al. (2007) have shown that gray matter development of the PFC of ADHD children and teens is delayed. Pharmacological targeting of BDNF itself is difficult, but treatment with a D3R agonist pre-pubertally may reduce behaviors that are associated with ADHD, including risk for SUD, changes in object recognition, and activity levels.

## Author contributions

Both Drs. Andersen and Sonntag contributed equally to the design, execution, and preparation of the manuscript.

### Conflict of interest statement

The authors declare that the research was conducted in the absence of any commercial or financial relationships that could be construed as a potential conflict of interest.
